# Upregulation of Mlxipl induced by cJun in the spinal dorsal horn after peripheral nerve injury counteracts mechanical allodynia by inhibiting neuroinflammation

**DOI:** 10.18632/aging.103313

**Published:** 2020-06-09

**Authors:** Hongrui Zhan, Yaping Wang, Shi Yu, Guiyuan Cai, Yanyan Zeng, Junqin Ma, Wei Liu, Wen Wu

**Affiliations:** 1 Department of Rehabilitation, Zhujiang Hospital, Southern Medical University, Guangzhou 510282, China; 2 Department of Rehabilitation, The Fifth Affiliated Hospital of Sun Yat-Sen University, Zhuhai 519000, Guangdong Province, China; 3 Guangdong Provincial Key Laboratory of Biomedical Imaging, The Fifth Affiliated Hospital of Sun Yat-Sen University, Zhuhai 519000, Guangdong Province, China; 4 Department of Rehabilitation, Shenzhen University General Hospital, Shenzhen 518055, China

**Keywords:** mechanical allodynia, neuroinflammation, microglia, Mlxipl, cJun

## Abstract

Mlxipl regulates glucose metabolism, lipogenesis and tumorigenesis and has a wide-ranging impact on human health and disease. However, the role of Mlxipl in neuropathic pain remains unknown. In this study, we found that Mlxipl was increased in the ipsilateral L4–L6 spinal dorsal horn after Spared Nerve Injury surgery. Knockdown of Mlxipl in the ipsilateral L4–L6 spinal dorsal horn by intraspinal microinjection aggravated Spared Nerve Injury-induced mechanical allodynia and inflammation in the spinal dorsal horn, on the contrary, overexpression of Mlxipl inhibited mechanical allodynia and inflammation. Subsequently, the rat Mlxipl promoter was analyzed using bioinformatics methods to predict the upstream transcription factor cJun. Luciferase assays and ChIP-qPCR confirmed that cJun bound to the promoter of Mlxipl and enhanced its expression. Finally, we demonstrated that Mlxipl inhibited the inflammatory responses of lipopolysaccharide-induced microglia and that Mlxipl was regulated by the transcription factor cJun. These findings suggested that cJun-induced Mlxipl upregulation in the spinal dorsal horn after peripheral nerve injury provided a protective mechanism for the development and progression of neuropathic pain by inhibiting microglial-derived neuroinflammation. Targeting Mlxipl in the spinal dorsal horn might represent an effective strategy for the treatment of neuropathic pain.

## INTRODUCTION

Neuropathic pain (NP) is a debilitating clinical condition caused by a lesion or disease of the somatosensory system. It is manifested as spontaneous persistent pain, allodynia or hyperalgesia [1]. NP has a very high prevalence (approximately 7–10%), and there is no satisfactory therapy due to its pathogenesis remains unclear. NP seriously affects the health and life quality and places a heavy burden on families and society [2]. Therefore, further exploration of the pathogenesis of NP might shine a light on the prevention and treatment of this disease.

Accumulating evidence suggests that neuroinflammation in the spinal dorsal horn (SDH) is involved in mechanical allodynia after peripheral nerve injury [3]. SDH is a key area that receives, processes and transmits peripheral nociceptive stimulation from dorsal root ganglion afferent. It is widely involved in central sensitization and participates in the development of chronic pain [1]. Microglia are resident immune cells of the central nervous system. Evidence suggests that microglia in the SDH play a vital role in the pathogenesis of NP [4, 5]. Microglia are activated after peripheral nerve injury, and then produce and release mediators that can modulate pain sensitivity [2, 5]. Mlxipl, aslo known as ChREBP, is a glucose-responsive transcription factor that was initially reported to regulate glucose metabolism, lipogenesis and tumorigenesis [6]. Recent study demonstrated that Mlxipl protects against atherosclerotic progression in an atherosclerotic model mouse by inhibiting lipopolysaccharide (LPS)-induced proinflammatory cytokines in macrophages [7]. Despite these findings, the role of Mlxipl in neuropathic pain remains poorly understood.

In this study, we hypothesized that activation of Mlxipl triggered by peripheral nerve injury inhibited inflammation in the SDH and thereby alleviated NP. First, we characterized whether the Mlxipl expression in the ipsilateral L4-L6 SDH was increased after Spared Nerve Injury (SNI) surgery. Subsequently, targeting down- or up-regulation of Mlxipl in the SDH were conducted to elucidate the role of Mlxipl in NP. Next, the upstream driver of Mlxipl was predicted using bioinformatics approach. Finally, the upstream transcription factors of Mlxipl were predicted and further validated by bioinformatics methods and experiments in vivo and in vitro.

## RESULTS

### Mlxipl was upregulated in ipsilateral SDH of SNI-induced mechanical allodynia rats

**Figure 1:**
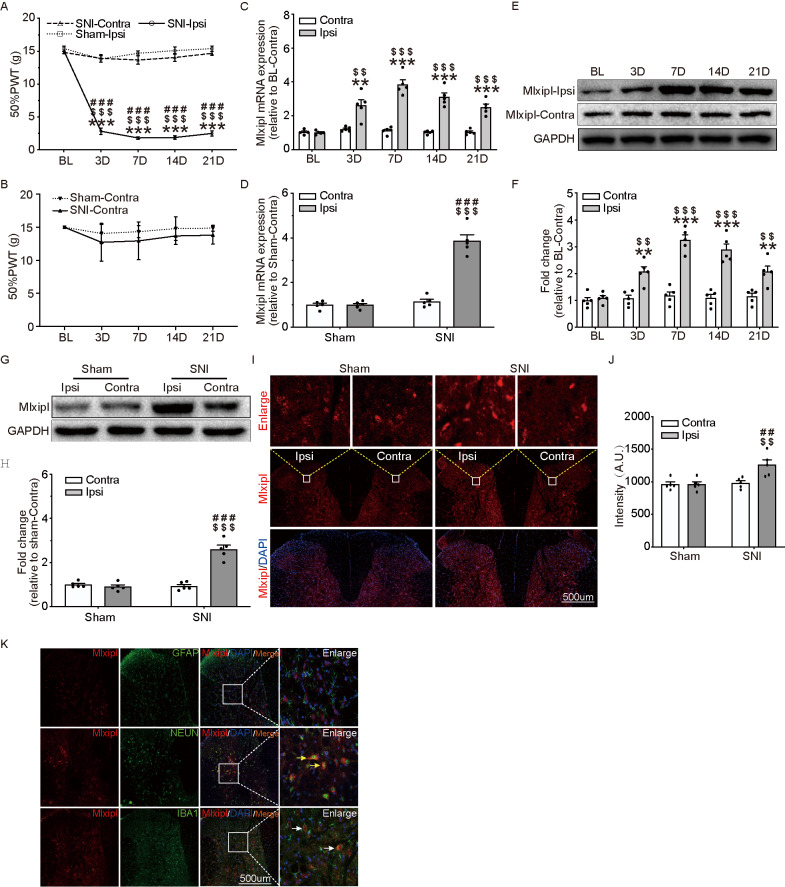
**Mlxipl was upregulated in ipsilateral SDH of SNI-induced mechanical allodynia rats.** (**A** and **B**) The Von Frey test were carried out before and after surgery (Sham or SNI). Mechanical allodynia was induced by SNI surgery in the ipsilateral paw. N = 5. ***P < 0.001 vs. SNI BL Ipsi; ^$$$^P < 0.001, ^$$^P < 0.01 vs. SNI Contra; ^###^P < 0.001 vs. Sham Ipsi. QPCR (**C**) and western blot (**E**) were performed before and after surgery. Mlxipl in the ipsilateral SDH was upregulated after SNI surgery. Quantification of the western blot (**F**). N = 5. ***P < 0.001, **P < 0.01 vs. SNI BL Ipsi; ^$$$^P < 0.001, ^$$^P < 0.01 vs. SNI BL Contra. QPCR (**D**), western blot (**G**) and immunofluorescence (**I**) were performed at day 7 after Sham or SNI surgery. Mlxipl was significantly upregulated in the ipsilateral SDH. Quantification of the western blot (**H**) and immunofluorescence (**J**). N = 5. ^$$$^P < 0.001, ^$$^P < 0.01 vs. SNI Contra; ^###^P < 0.001, ^##^P < 0.01 vs. Sham Ipsi. (**K**) Double immunofluorescence staining showed that Mlxipl was co-localized with Iba1 (white arrows) and NeuN (yellow arrows) but not GFAP. BL, baseline (before surgery); ipsi, ipsilateral; Contra, contralateral; SNI, spare nerve injury; SDH, spinal dorsal horn.

In order to verify that the SNI surgery induced mechanical allodynia, the 50% paw withdraw threshold (50% PWT) of behavior test were examined before and after sham or SNI surgery. The ipsilateral 50% PWT was remarkably elevated after SNI surgery (Figure 1A), while no obvious difference was observed in contralateral side (Figure 1B). Additionally, no noticeable difference existed between contralateral side and the ipsilateral side in Sham group.

Mlxipl expression in the L4–L6 SDH were detected after sham or SNI surgery by qPCR, western blot and immunofluorescence assays. Compared with preoperative baseline (BL), the Mlxipl mRNA and protein expression in the ipsilateral SDH were manifestly activated after SNI surgery. By contrast, a negligible amount of Mlxipl alteration was produced in the contralateral SDH (Figure 1C, 1E and 1F). Consistently, qPCR, western blot and immunofluorescence revealed that Mlxipl expression was apparently augmented at day 7 after SNI surgery in the ipsilateral SDH, but not the contralateral side (Figure 1D and 1G–1J).

In order to further investigate the cellular localization of Mlxipl in the SDH, double immunofluorescence staining was implemented on Mlxipl with cell-specific markers, NeuN (neurons), Iba1 (microglia) or GFAP (astrocytes), respectively. The results displayed that Mlxipl was co-localized with Iba1 and NeuN but not GFAP. These data implied that Mlxipl played a crucial role in NP through microglia and neurons following SNI surgery (Figure 1K).

### Knockdown of Mlxipl in the SDH by intraspinal microinjection aggravated mechanical allodynia and neuroinflammation

**Figure 2:**
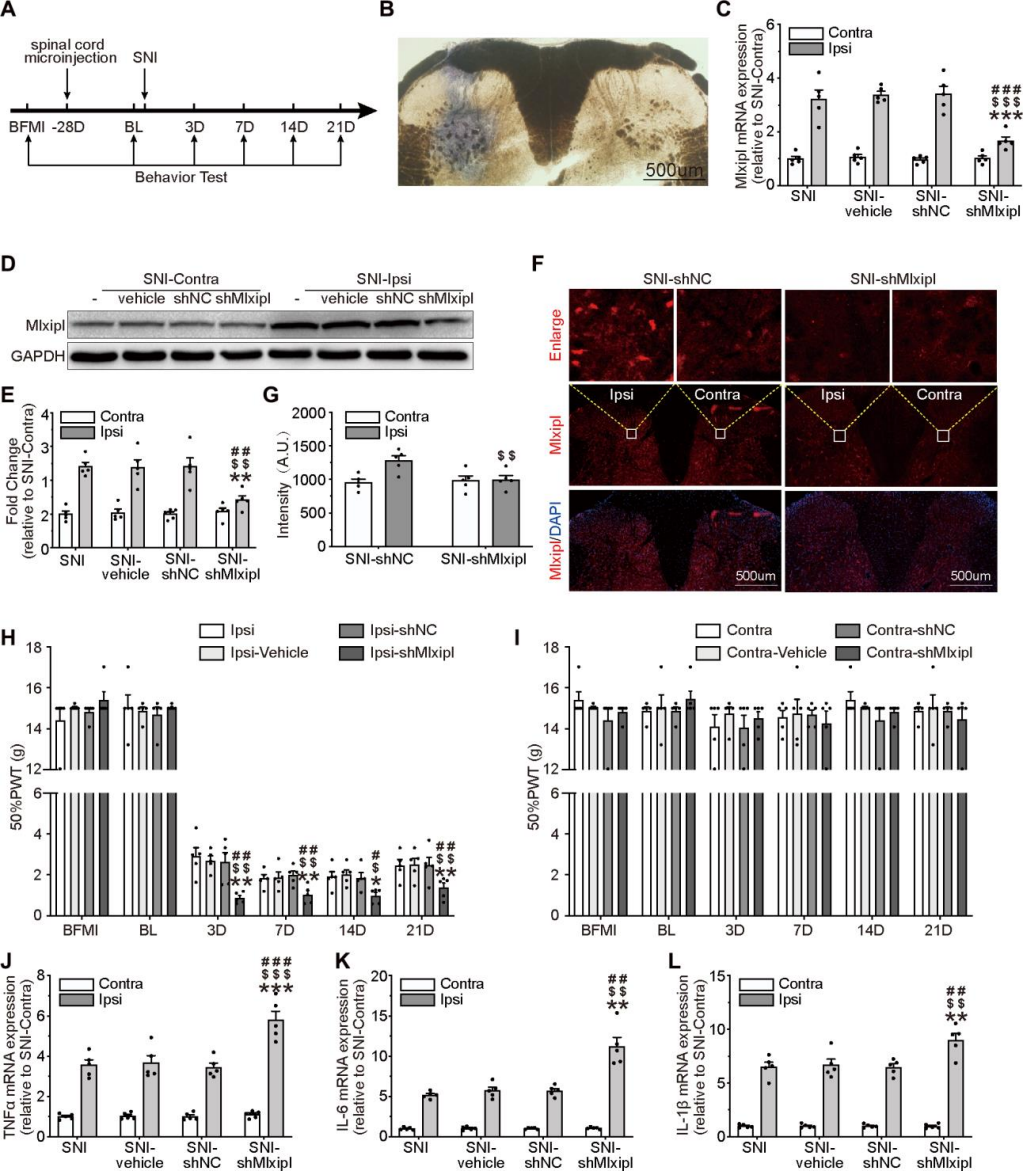
**Knockdown of Mlxipl in the SDH promoted mechanical allodynia and neuroinflammation.** (**A**) A schematic that illustrates the timing of the main experimental procedures. (**B**) The injection location was confirmed by intraspinal microinjection of trypan blue. (**C**–**G**) Mlxipl in the ipsilateral SDH was knockdown by intraspinal microinjection of shMLXIPL. SNI surgery was performed on day 28 after intraspinal microinjection. QPCR (**C**), western blot (**D**) and immunofluorescence (**F**) were performed at day 7 after SNI surgery. Quantification of western blot (E) and immunofluorescence (**G**). N = 5. ***P < 0.001, **P < 0.01 vs. SNI Ipsi; ^###^P < 0.001, ^##^P < 0.01 vs. SNI vehicle Ipsi; ^$$$^P < 0.001, ^$$^P < 0.01 vs. SNI shNC Ipsi. (**H** and **I**) Knockdown of Mlxipl promoted mechanical allodynia in the ipsilateral paw. The Von Frey test was performed before and after SNI surgery with or without pre-microinjection. N = 5. **P < 0.01, *P < 0.05 vs. SNI Ipsi. ^##^P < 0.01, ^#^P < 0.05 vs. SNI vehicle Ipsi; ^$$^P < 0.01, ^$^P < 0.05 vs. SNI shNC Ipsi. (**J**–**L**) Knockdown of Mlxipl promoted neuroinflammation in the ipsilateral SDH. Proinflammatory cytokines were detected using qPCR at day 7 after SNI surgery. N = 5. ***P < 0.001 vs. SNI Ipsi; ^###^P < 0.001 vs. SNI vehicle Ipsi; ^$$$^P < 0.001 vs. SNI shNC Ipsi. BFMI, before microinjection; BL, baseline (before surgery); ipsi, ipsilateral; Contra, contralateral; SNI, spare nerve injury; SDH, spinal dorsal horn.

To clarify whether the upregulation of Mlxipl in the ipsilateral L4–L6 SDH was related to mechanical allodynia and neuroinflammation, intraspinal microinjection of interfering Mlxipl adeno-associated virus (shMlxipl) 28 days prior to SNI surgery was performed to knock down the Mlxipl expression. Multiple assays, including qPCR, western blot and immunofluorescence were conducted at day7 after SNI surgery. A scrambled adeno-associated virus (shNC) was used as a control. Figure 2A illustrates the timing of the main experimental procedures. The injection location was verified by intraspinal microinjection of trypan blue (Figure 2B).

Mlxipl mRNA and protein were obviously suppressed after intraspinal microinjection of shMlxipl in the ipsilateral SDH, without influencing the Mlxipl expression in the contralateral SDH (Figure 2C–2G).

To clarify the relationship between Mlxipl and mechanical allodynia, the 50% PWT was tested before and after microinjection. The ipsilateral 50% PWT of the shMlxipl group was lower at day 3, 7, 14 and 21 after SNI surgery than the other groups. In other words, knockdown of Mlxipl in the ipsilateral SDH aggravated mechanical allodynia (Figure 2H). Surprisingly, microinjection of AAV-shMlxipl did not affect the mechanical allodynia on either side with regard to basic nociception, as no significant change was shown in the 50% PWT on baseline (before SNI surgery) (Figure 2H–2I).

QPCR analysis was applied to probe the effect of Mlxipl knockdown on proinflammatory cytokines IL-1β, IL-6 and TNF-α. Compared with other groups, the levels of proinflammatory cytokines in the ipsilateral SDH of shMlxipl group significantly raised at day 7 after SNI surgery. However, no remarkable difference was found in the contralateral SDH among all groups (Figure 2J–2L). In summary, knockdown of Mlxipl in the SDH by intraspinal microinjection promoted mechanical allodynia and neuroinflammation.

### Overexpression of Mlxipl in the SDH by intraspinal microinjection inhibited mechanical allodynia and neuroinflammation

**Figure 3:**
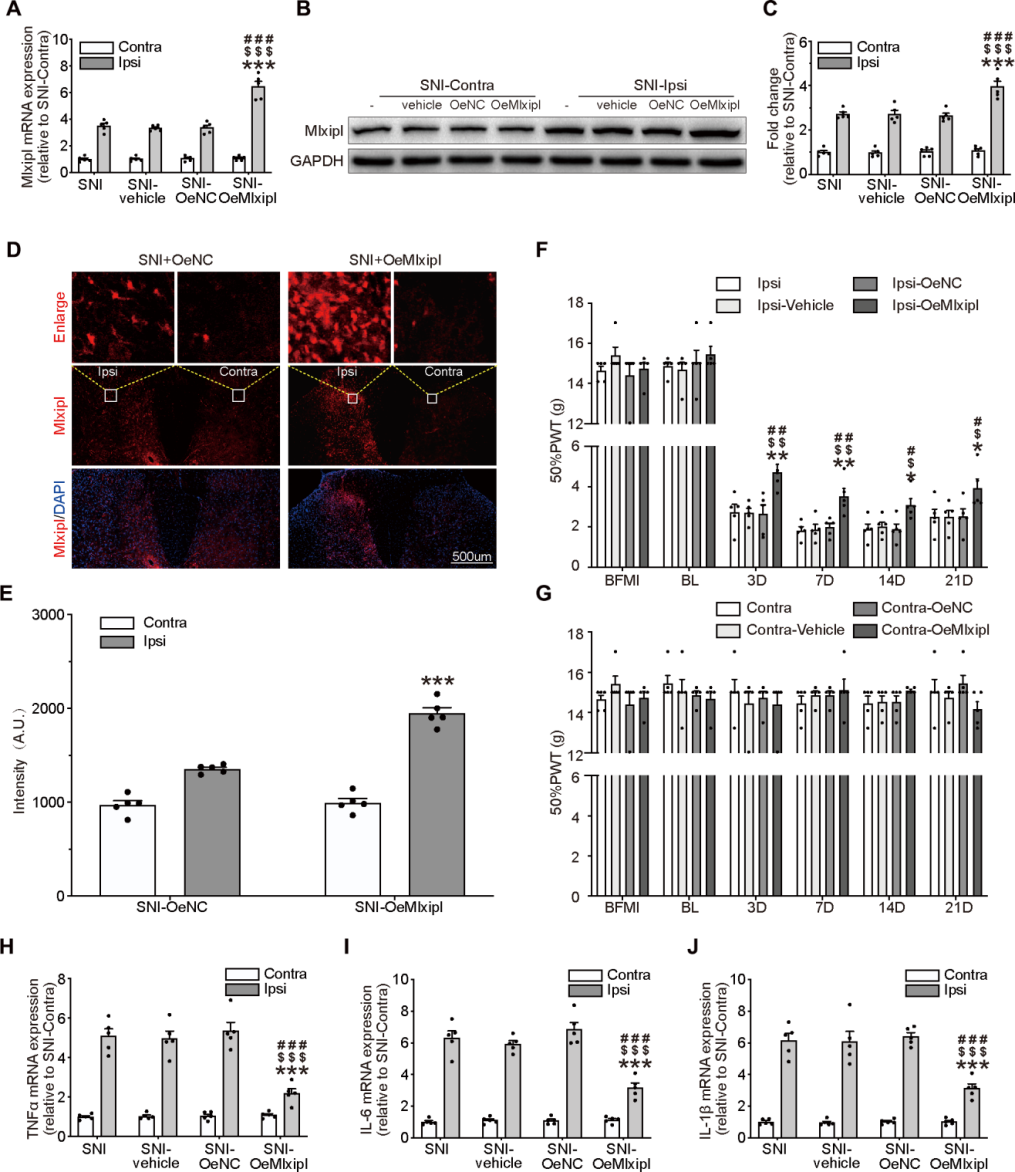
**Overexpression of Mlxipl in the SDH inhibited mechanical allodynia and neuroinflammation.** (**A**–**E**) Mlxipl in the ipsilateral SDH was overexpressed by intraspinal microinjection of OeMLXIPL. SNI surgery was performed on day 28 after intraspinal microinjection. QPCR (**A**), western blot (**B**) and immunofluorescence (**D**) were performed at day 7 after SNI surgery. Quantification of western blot (C) and immunofluorescence (**E**). N = 5. ***P < 0.001 vs. SNI Ipsi; ^###^P < 0.001 vs. SNI vehicle Ipsi; ^$$$^P < 0.001 vs. SNI shNC Ipsi. (**F** and **G**) Overexpression of Mlxipl inhibited mechanical allodynia in the ipsilateral paw. The Von Frey test was performed before and after SNI surgery with or without pre-microinjection. N = 5. **P < 0.01, *P < 0.05 vs. SNI Ipsi 0D; ^##^P < 0.01, ^#^P < 0.05 vs. SNI vehicle Ipsi 0D; ^$$^P < 0.01, ^$^P < 0.05 vs. SNI OeNC Ipsi 0D. (**H**–**J**) Overexpression of Mlxipl inhibited neuroinflammation in the ipsilateral SDH. Proinflammatory cytokines were detected using qPCR at day 7 after SNI surgery. N = 5. ***P < 0.001 vs. SNI Ipsi; ^###^P < 0.001 vs. SNI vehicle Ipsi; ^$$$^P < 0.001 vs. SNI shNC Ipsi. BFMI, before microinjection; BL, baseline (before surgery); ipsi, ipsilateral; Contra, contralateral; SNI, spare nerve injury; SDH, spinal dorsal horn.

To further explore the role of Mlxipl in neuropathic pain, intraspinal microinjection of adeno-associated virus encoding Mlxipl (OeMlxipl) 28 days prior to SNI surgery was implemented to upregulate the Mlxipl expression. Mlxipl in the SDH was examined on day 7 after SNI surgery using qPCR, western blot and immunofluorescence. Mlxipl mRNA and protein expression in the ipsilateral SDH of the OeMlxipl group were obviously upregulated at day 7 after SNI surgery (Figure 3A–3E). Similar to the results of shMlxipl microinjection, microinjection of OeMlxipl had no effect on Mlxipl expression in the contralateral side.

The ipsilateral 50% PWT of the OeMlxipl group was markedly higher at day 3, 7, 14 and 21 after SNI surgery than other groups (Figure 3F). There was no significant change in the 50% PWT on the baseline, suggesting that Mlxipl overexpression did not affect the basic nociception (Figure 3F–3G).

The qPCR analysis of proinflammatory cytokines was implemented to validate the alleviation of inflammation by Mlxipl's overexpression. The results showed that the levels of proinflammatory cytokines in the ipsilateral SDH were significantly decrease in presence of Mlxipl upregulation, which supported the results that Mlxipl impaired inflammation response (Figure 3H–3J). As anticipated, no discernible differences in inflammation response were detected in the contralateral SDH (Figure 3H–3J). In short, overexpression of Mlxipl in the SDH inhibited mechanical allodynia and neuroinflammation.

### cJun directly promoted Mlxipl expression at the transcriptional level

To investigate the causes of Mlxipl upregulation at the transcriptional level after peripheral nerve injury, three published bioinformatics sites JASPAR (http://jaspar.genereg.net/) [8], PROMO (http://alggen.lsi.upc.es/) [9, 10], and GTRD (http://gtrd.biouml.org/) [11] were used to predict the potential transcription factors. CJun and SP1 were predicted to bind to promoter region (2000 base pairs upstream of the transcription starting site) of Mlxipl. CJun was further studied with the highest score among the three prediction methods (Supplementary Table 4). Besides, knockdown of SP1 was not significantly inhibited the mRNA and protein expression of Mlxipl (Supplementary Figure 1).

**Figure 4:**
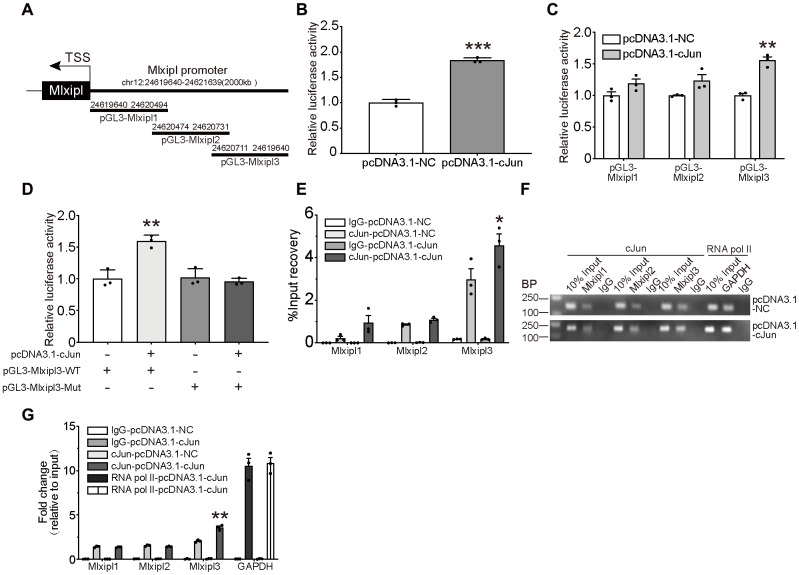
**cJun directly regulated Mlxipl expression at the transcriptional level.** (**A**) Schematic diagrams showed the Mlxipl promoter and its fragments containing predicting binding sites. The diagrams show the Mlxipl promoter and its fragments, which were used to predict potential binding sites. (**B**) Luciferase reporter assays of full-length Mlxipl reporter. PcDNA3.1-cJun or pcDNA3.1-NC was co-transfected with full-length Mlxipl reporter in microglia. PcDNA3.1-cJun promoted luciferase activity of full-length Mlxipl reporter luciferase activity. The data were relative to the pcDNA3.1-NC group. N = 3. ***P < 0.001 vs. pcDNA3.1-NC. (**C**) Luciferase reporter assays of fragments of Mlxipl reporter. PcDNA3.1-cJun or pcDNA3.1-NC was co-transfected with fragmented Mlxipl promoters (pGL3-Mlxipl1, pGL3-Mlxipl2 and pGL3-Mlxipl3), respectively. PcDNA3.1-cJun promoted luciferase activity of pGL3-Mlxipl3 reporter luciferase activity. N = 3. **P < 0.01 vs. pcDNA3.1-NC. (**D**) Luciferase reporter assays of mutation of pGL3-Mlxipl3 reporter. PcDNA3.1-cJun or pcDNA3.1-NC was co-transfected with pGL3-Mlxipl3 reporter with or without mutation. N = 3. **P < 0.01 vs. pcDNA3.1-cJun+pGL3-Mlxipl3. (**E** and **F**) ChIP assays with anti-cJun antibody were performed followed by qPCR or agarose gel electrophoresis. The DNA product of ChIP was detected by fragmented Mlxipl promoter primers. PcDAN3.1-cJun transfection enriched more Mlxipl3 fragments. IgG was used as a negative control; RNA pol II was used as a positive control system; ChIP DNA mixture was used as Input, and the sample loading was 10% of other groups. Quantification of agarose gel electrophoresis (**G**). N= 3. *P < 0.05, **P < 0.01 vs. pcDNA3.1-NC. TSS, transcription starting site.

As shown in the schematic diagram (Figure 4A), we cloned the full-length sequence of Mlxipl promoter into a luciferase reporter plasmid (pGL3-Mlxipl) and constructed a cJun expression plasmid (pcDNA3.1-Jun). Primary microglia were co-transfected with the pGL3-Mlxipl and pcDNA3.1-Jun plasmids to perform the dual-luciferase assays. The Mlxipl reporter luciferase activities of the pcDNA3.1-cJun group were strikingly higher than those of the pcDNA3.1-NC group, suggesting that cJun enhanced the transcription of Mlxipl (Figure 4B).

To further clarify the binding of cJun to the Mlxipl promoter, three candidate cJun binding sites were obtained by bioinformatics approaches: nucleotides (nt) 24,620,786–24,621,639; 24,620,549–24,620,805 and 24,619,640–24,620,568. The full-length sequence of Mlxipl promoter was then fragmented into overlapping fragments with 20 base pairs for these three regions, namely Mlxipl1, Mlxipl2 and Mlxipl3. The luciferase reporters pGL3-Mlxipl1, pGL3-Mlxipl2 and pGL3-Mlxipl3 were constructed and co-transfected with pcDNA3.1-NC or pcDNA3.1-cJun, respectively. The results demonstrated that pGL3-Mlxipl3 luciferase activities of the pcDNA3.1-cJun group were noticeably greater than those of the pcDNA3.1-NC group. No intergroup difference (pcDNA3.1-NC group versus pcDNA3.1-cJun group) was occurred in the luciferase activities of pGL3-Mlxipl1 and pGL3-Mlxipl2 (Figure 4C). Subsequently, luciferase assays of mutation of pGL3-Mlxipl3 promoter indicated that the luciferase activities of the pGL3-Mlxipl3-Mut group strongly declined in comparison to the pGL3-Mlxipl3-WT group (Figure 4D).

To further confirm the binding between cJun and Mlxipl promoter, Chromatin Immunoprecipitation (ChIP) coupled with quantitative PCR (ChIP-qPCR) or agarose gel electrophoresis were conducted to quantify the enrichment of Mlxipl1, Mlxipl2 or Mlxipl3. ChIP using anti-cJun antibody enriched more Mlxipl3 in the pcDNA3.1-cJun group by comparison to the pcDNA3.1-NC group. Mlxipl1 or Mlxipl2 did not show statistically significant differences between the pcDNA3.1-cJun groups and pcDNA3.1-NC group (Figure 4E–4G). Accordingly, these results confirmed that cJun directly promoted Mlxipl transcription by binding to transcript factor binding site 3 of Mlxipl promoter.

### Mlxipl reversed the neuroinflammation and mechanical allodynia induced by cJun

**Figure 5:**
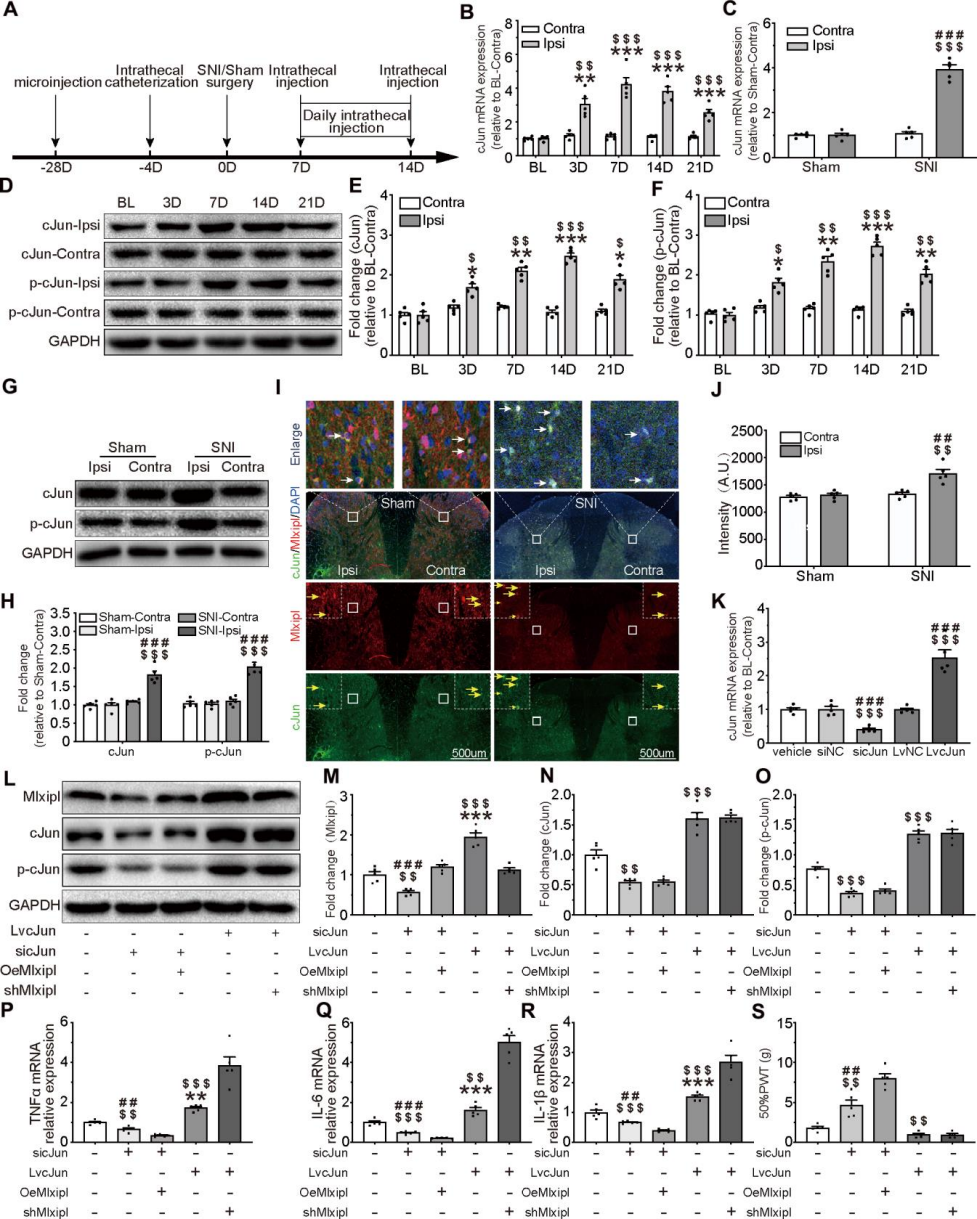
**cJun-induced Mlxipl upregulation protectively inhibited inflammation in the SDH and improved mechanical allodynia.** (**A**) The schematic illustrates the timing of the main experimental process. (**B** and **D**–**F**) QPCR (**B**) and western blot (**D**) were performed before and after surgery. CJun or p-cJun in the ipsilateral SDH was upregulated after SNI surgery. Quantification of the western blot (E-F). N = 5. ***P < 0.001, **P < 0.01, *P < 0.05 vs. SNI BL Ipsi; ^$$$^P < 0.001, ^$$^P < 0.01, ^$^P < 0.05 vs. SNI BL Contra. (**C** and **G**–**J**) QPCR(**C**), western blot (**G**) and immunofluorescence (**I**) were performed at day 7 after Sham or SNI surgery. CJun or p-cJun was significantly upregulated in the ipsilateral SDH. cJun was co-localized with Mlxipl (white arrows). Quantification of the western blot (**H**) and immunofluorescence (**J**). N = 5. ^$$$^P < 0.001, ^$$^P < 0.01 vs. SNI Contra; ^###^P < 0.001, ^##^P < 0.01 vs. Sham Ipsi. (**K**–**O**) Intrathecal injections of cJun small interfering RNA (sicJun) or lentivirus encoding cJun (LvcJun) were performed at day 7 after SNI surgery. The injections were carried out once a day for 7 consecutive days and then cJun expression was detected by qPCR (**K**) and western blot (**L**). Knockdown of cJun inhibited the expression of Mlxipl and p-cJun. Overexpression of cJun promoted the expression of Mlxipl and p-cJun (**L**-**O**). Quantification of the western blots (**M**–**O**). N = 5. ^$$$^P < 0.001, ^$$^P < 0.01 vs. vehicle; ^###^P < 0.001, ^##^P < 0.01 vs. sicJun+OeMlxipl; ***P < 0.001, ^**^P < 0.01 vs. LvcJun+shMlxipl. (**P**–**S**) The effect of cJun on neuroinflammation (**P**–**R**) and mechanical allodynia (**S**), with or without the pre-microinjection of OeMlxipl or shMlxipl. N = 5. ^$$$^P < 0.001, ^$$^P < 0.01 vs. vehicle; ^###^P < 0.001, ^##^P < 0.01 vs. sicJun+OeMlxipl; ***P < 0.001, ^**^P < 0.01 vs. LvcJun+shMlxipl. BL, baseline (before surgery); ipsi, ipsilateral; Contra, contralateral; SNI, spare nerve injury; SDH, spinal dorsal horn.

To evaluate the effect of cJun on mechanical allodynia after peripheral nerve injury, the cJun expression in the SDH was analyzed with qPCR, western blot and immunofluorescence. Compared with pre-operative baseline, the cJun and phosphorylated cJun (p-cJun) expression were dramatically increased post-SNI surgery, while no obvious difference was observed in contralateral side (Figure 5B and 5D–5F). These results were in accord with the results of cJun expression obtained at day 7 after SNI surgery. No significant changes were observed in the contralateral side of all groups (Figure 5C and 5G–5J). As expected, immunofluorescence double staining showed that cJun was co-localized with Mlxipl (Figure 5I).

To further probe whether cJun triggered Mlxipl transcription *in vivo*, intrathecal injections of cJun small interfering RNA (sicJun) or lentivirus encoding cJun (LvcJun) were performed at day 7 after SNI surgery. The results of qPCR and western blot revealed that cJun was significantly downregulated by intrathecal injections of sicJun and upregulated by intrathecal injections of LvcJun (Figure 5K–5O). Of note, knockdown of cJun hindered the expression of Mlxipl and p-cJun. In contrary, overexpression of cJun escalated the expression of Mlxipl and p-cJun (Figure 5L–5O). These data fully confirmed that cJun promoted Mlxipl in the SDH after SNI surgery.

An opposite trend was observed in behavioral testing, which illustrated that 50% PWT elevated in the sicJun group and diminished in the LvcJun group (Figure 5S). Furthermore, the results of the proinflammation cytokines demonstrated that knockdown of cJun inhibited the inflammation response while overexpression of cJun promoted inflammation response in the SDH. These results suggested that cJun promoted neuroinflammation and mechanical allodynia. Furthermore, the results of the proinflammation cytokines showed that knockdown of cJun inhibited the inflammation response while overexpression of cJun promoted inflammation response in the SDH. These results suggested that cJun promoted neuroinflammation and mechanical allodynia.

To further clarify the relationship between the cJun, Mlxipl, inflammation and mechanical allodynia, we performed pre-microinjection of OeMlxipl with intrathecal injection of sicJun or pre-microinjection of shMlxipl with intrathecal injection of LvcJun. Figure 5A illustrated the timing of the main experiment procedures. Notably, pre-microinjection of OeMlxipl with intrathecal injection of sicJun improved inflammation and mechanical allodynia to a greater extent compared with intrathecal injection of sicJun alone (Figure 5P–5S). Similarly, pre-microinjection of shMlxipl with intrathecal injection of LvcJun significantly aggravated inflammation and mechanical allodynia to a greater extent compared with intrathecal injection of LvcJun alone (Figure 5P–5S). Unexpectedly, compared with LvcJun group, shMlxipl-LvcJun group did not significantly change in mechanical allodynia (data not shown).

Overall, these data indicated that cJun was upregulated after SNI surgery, which aggravated the neuroinflammation and mechanical allodynia. But on the other hand, cJun activated Mlxipl transcription and thus hampered neuroinflammation and mechanical allodynia. In general, however, the pro-inflammatory effect of cJun was superior to the Mlxipl-mediated effect on inhibition.

### Mlxipl inhibited the cJun-mediated inflammatory response in microglia

**Figure 6:**
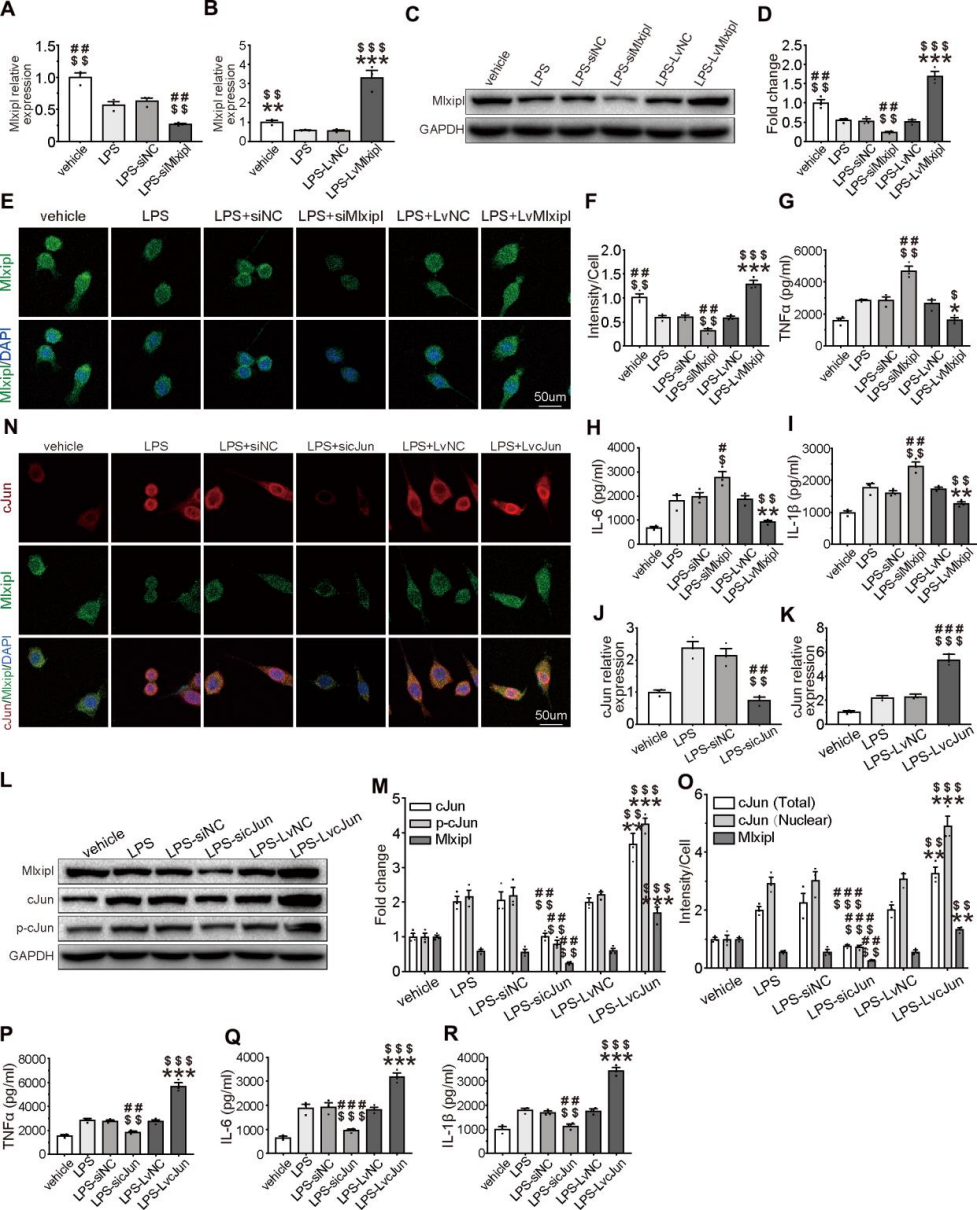
**Mlxipl inhibited the cJun-mediated inflammatory response in microglia.** (**A**–**F**) The Mlxipl expression was detected by QPCR (**A**–**B**), western blot (**E**) and immunofluorescence (**E**). The primary microglia were co-transfected with siMlxipl or LvMlxipl for 48 hr, and then treated with LPS (1 μg/ml) for 24 hr. Quantification of the western blot (**D**) and immunofluorescence (**F**). Data relative to vehicle. N = 3. ^$$$^P < 0.001, ^$$^P < 0.01, ^$^P < 0.05 vs. LPS; ^###^P < 0.001, ^##^P < 0.01 vs. LPS-siNC; ***P < 0.001, **P < 0.01, *P < 0.05 vs. LPS-LvNC. (**G**–**I**) ELISA were performed to detect the expression of proinflammation cytokines. Mlxipl inhibited inflammation response in LPS-induced microglia. N = 3. ^$$$^P < 0.001, ^$$^P < 0.01, ^$^P < 0.05 vs. LPS; ^###^P < 0.001, ^##^P < 0.01 vs. LPS-siNC; ***P < 0.001, **P < 0.01, *P < 0.05 vs. LPS-LvNC. (**J**–**O**) The cJun expression was detected by QPCR (**J–K**), western blot (**L**) and immunofluorescence (**N**). The primary microglia were co-transfected with sicJun or LvcJun for 48 hr, and then treated with LPS (1 μg/ml) for 24 hr. Knockdown of cJun inhibited the expression of Mlxipl and p-cJun. Overexpression of cJun promoted the expression of Mlxipl and p-cJun. Quantification of the western blot (**M**) and immunofluorescence (**O**). Data relative to vehicle. N = 3. ^$$$^P < 0.001, ^$$^P < 0.01, ^$^P < 0.05 vs. LPS; ^###^P < 0.001, ^##^P < 0.01 vs. LPS-siNC; ***P < 0.001, **P < 0.01, *P < 0.05 vs. LPS-LvNC. (**P**–**R**) ELISA were performed to detect the expression of proinflammation cytokines. CJun promoted inflammation response in LPS-induced microglia. N = 3. ^$$$^P < 0.001, ^$$^P < 0.01, ^$^P < 0.05 vs. LPS; ^###^P < 0.001, ^##^P < 0.01 vs. LPS-siNC; ***P < 0.001, **P < 0.01, *P < 0.05 vs. LPS-LvNC.

To better characterize the inhibitory effect of Mlxipl on inflammation, Mlxipl was knocked down or overexpressed in LPS-induced primary microglia. The primary microglia were treated with LPS (1 μg/ml) for 24 hr. The results exhibited that Mlxipl was notably impeded in the LPS group in comparison with the vehicle group (Figure 6A–6F). The administration of siMlxipl abated Mlxipl expression. On the contrary, the administration of LvMlxipl advanced the Mlxipl expression (Figure 6A–6F). It was of critical importance that overexpression of Mlxipl significantly inhibited the LPS-induced inflammatory response, whereas knockdown of Mlxipl significantly promoted the LPS-induced inflammatory response (Figure 6G–6I). These results fully confirmed that Mlxipl restrained the LPS-induced inflammation.

In order to ascertain that cJun stimulated Mlxipl transcription in microglia, cJun was knocked down or overexpressed by transfection of sicJun or LvcJun in LPS-induced primary microglia. QPCR, western blot and immunofluorescence analysis revealed that an increase in Mlxipl was observed with an increase in cJun. Also, a decrease in Mlxipl was found with a decrease in cJun (Figure 6J–6O). Additionally, western blot analysis of primary microglia treated with T-5224 (a cJun inhibitor) for 24 hr indicated that T5224 dramatically restrained Mlxipl pression (Supplementary Figure 2).

Furthermore, ELISA detection demonstrates that overexpression of cJun enormously stimulated the LPS-induced inflammatory response. in sharp contrast, downregulation of cJun obviously hindered the LPS-induced inflammatory response (Figure 6P–6R). In short, cJun promoted LPS-induced inflammation response. In summary, these results confirmed that although cJun promoted Mlxipl expression and inflammation response, Mlxipl in turn inhibited the inflammatory response. Overall, the pro-inflammatory effect of cJun was superior to the Mlxipl-mediated inhibitory effect.

## DISCUSSION

**Figure 7:**
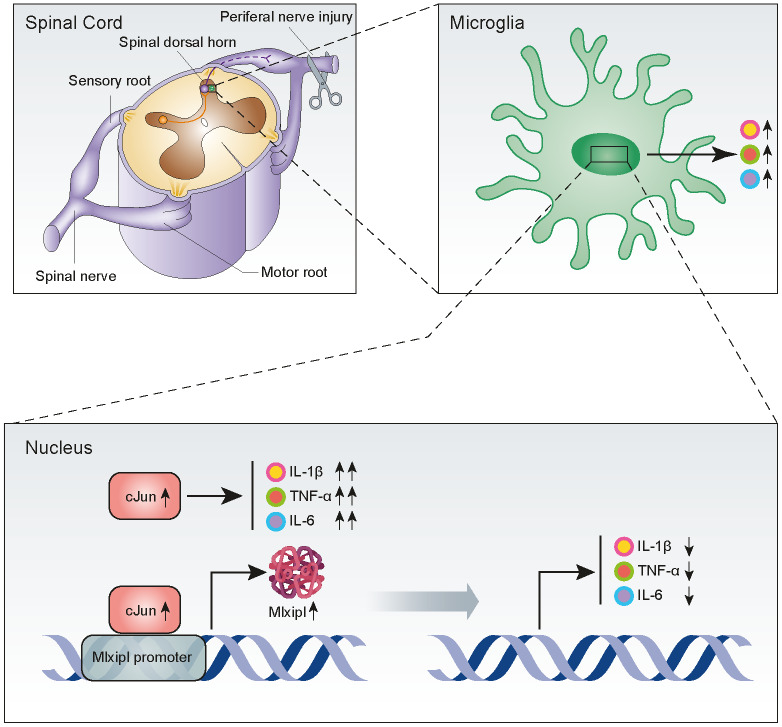
**Upregulation of Mlxipl induced by cJun in the spinal dorsal horn after peripheral nerve injury counteracts mechanical allodynia by inhibiting neuroinflammation.** CJun expression in the SDH was upregulated after peripheral nerve injury, and this increased Mlxipl transcription. Upregulation of Mlxipl counteracts mechanical allodynia by inhibiting neuroinflammation derived from microglia in the SDH.

Mechanical allodynia caused by SNI is widely used to simulate NP induced by clinical nerve trauma [12]. Exploring the underlying mechanisms of NP should provide new therapeutic strategies for its prevention and treatment. In this study, we reported that cJun-induced upregulation of Mlxipl in the ipsilateral SDH inhibited the inflammation and mechanical allodynia after SNI surgery (Figure 7). These findings suggested that Mlxipl might be a potential target for the prevention and treatment of NP.

Mechanical allodynia is caused by damaged nerve fibers that send abnormal signals to the pain conduction pathway [1]. In the neural circuit, the SDH, a vital region for peripheral sensory information input and supraspinal descending signal output, plays a critical role in neuropathic pain. However, its mechanism is still not entirely clear [13]. Stereotactic microinjection technology for the delivery of vectors is a valuable method in neuroscience research [14]. The method accurately targets the region of interest and stably manipulate the gene in the target region. It allows for excellent spatiotemporal control of gene expression [15]. In this study, this method accurately manipulated Mlxipl expression in the ipsilateral L4–L6 SDH to provide stable and reliable results for exploring the neuropathic pain mechanism. In the first part of the study, we found that Mlxipl was upregulated in the ipsilateral SDH of SNI-induced mechanical allodynia rats. These results were consistent with a previous study by Zhou et al. [16], which reported that Mlxipl expression in the L4–L5 spinal cord was significantly increased on day 7 after SNI (using high-throughput sequencing).

To date, research on the Mlxipl protein has focused on lipogenesis, glucose metabolism, regulation of inflammation and tumorigenesis [6]. There is no further study on whether Mlxipl in the spinal cord level is involved in mechanical allodynia. This study found that Mlxipl in the ipsilateral L4–L6 SDH increased after SNI surgery, suggesting that Mlxipl was involved in the pathogenesis of neuropathic pain. Subsequently, Mlxipl in the ipsilateral L4–L6 SDH was knocked down by intraspinal microinjection of AAV-shMlxipl and overexpressed by intraspinal microinjection of AAV-OeMlxipl. It’s worth noting that knockdown of Mlxipl promoted mechanical allodynia, while overexpression of Mlxipl inhibited mechanical allodynia. Our findings are the first to report the critical role of Mlxipl in the pathogenesis of NP.

Neuroinflammation is one of the most important theories of the pathogenesis of neuropathic pain [5, 17, 18]. In this study, knockdown of Mlxipl promoted inflammation response in the SDH, while overexpression of Mlxipl inhibited inflammation response in the SDH. These results are consistent with a previous word reported by Sarrazy et al. [7]. It was reported that Mlxipl in peripheral macrophages offers protection against atherosclerotic processes in atherosclerotic mice. Mlxipl exerted its anti-inflammatory effects in LPS-induced macrophage activation by inhibiting the phosphorylation of NF-κB subunit p65 [7]. Interestingly, Mlxipl has been reported to aggravate diabetic nephropathy by pro-inflammatory effects in a streptozotocin (STZ)-induced diabetic mice model [19]. We speculate that there are a few reasons for the differential Mlxipl-mediated regulation of inflammation. First and foremost, Mlxipl protein functions through distinct pathways to regulate inflammation in different diseases and phenotypes. The central nervous system exhibits high specificity, and thus its inflammatory response is not equivalent to that of the peripheral system [20]. Second, the inflammatory response in the ipsilateral SDH after peripheral nerve injury is regulated by various factors [21, 22]. Our study indicated that Mlxipl was activated after peripheral nerve injury, and this activation might be related to various factors such as transcription factor triggering, epigenetic modification, post-translational modification and increased RNA stability after peripheral axon injury [23].

Previous studies demonstrated that transcriptional dependence is one of the critical features of peripheral axonal injury regeneration, and many transcription factors are directly activated downstream to the injury signaling pathway [24].

In this study, the transcription factors of Mlxipl were predicted by bioinformatics methods. Luciferase assays and ChIP-qPCR were performed to confirm that cJun promoted the expression of Mlxipl at transcriptional level. CJun is a key component of the heterodimeric AP-1 transcription factor and is highly expressed in response to neuronal damage. CJun plays an important role in dominating neural cell death and degeneration, promoting gliosis and inflammation as well as neural plasticity and repair [25, 26]. One of the core physiological and pathological changes induced by peripheral nerve injury is the rapid induction, continuous expression and activation of cJun [27].

Previous studies demonstrated a significant pro-inflammatory effect of cJun activation in microglia [28, 29], consistent with what we reported here that cJun promoted inflammation after peripheral nerve injury. On one hand, this study demonstrated that cJun activation in the SDH directly promoted inflammation. On the other hand, cJun promoted Mlxipl transcription, and the upregulation of Mlxipl in turn inhibited the inflammatory response in the SDH. This study reported for the first time that cJun-induced Mlxipl inhibited neuroinflammation and mechanical allodynia after peripheral nerve injury. We propose a new insight into the protective mechanism of Mlxipl in NP induced by peripheral nerve injury. The current findings broaden our insights into the self-protection mechanisms of NP caused by peripheral nerve injury. This protective mechanism partially prevents the inflammatory response in the SDH from being over-amplified after peripheral nerve injury.

Notably, previous studies have shown that cJun expression in neurons is maintained at a high level after peripheral nerve section or crush [30, 31]. In this study, Mlxipl protein in the SDH was co-localized with neurons and microglia. We speculate that—in addition to inhibiting the inflammatory response of the dorsal spinal cord—Mlxipl might exert anti-neuropathic pain effects by a potential mechanism that directly affects the electrophysiological activity of neurons. These possibilities will be further investigated in our future research through electrophysiological techniques such as patch clamps. In addition, in order to regulate cJun expression in the SDH, we used intrathecal injection to regulate its expression. Although this method can modulate the expression of cJun in the SDH, the effect is not limited to ipsilateral SDH: it still has a certain effect on the expression of cJun in the entire spinal cord. In future studies, we will perform targeted cJun knockout to further investigate the cJun-mediated regulation of Mlxipl and its contribution to neuropathic pain.

## CONCLUSION

Our findings demonstrated that cJun-induced Mlxipl upregulation in the SDH after peripheral nerve injury exerted an anti-neuropathic pain effect by inhibiting the microglia-derived inflammatory response. Mlxipl provided a protective mechanism for the development and progression of NP by inhibiting neuroinflammation in the SDH. Mlxipl in the SDH might be a new target and effective strategy for the treatment of NP.

## MATERIALS AND METHODS

### Animals

Male Sprague Dawley rats (200–250 g) were purchased from the Experimental Animal Center of Southern Medical University. The rats were housed on a 12-h light-dark cycle at room temperature (23 ± 1°C), with 50% relative humidity and free access to rodent feed and water. All surgical and experimental procedures in this study were approved by the Southern Medical University Animal Care and Use Committee (Ethics No. L2019238) and followed the guidelines of the National Institutes of Health on Animal Care and Ethics. Every effort was made to minimise any pain or suffering of the animals. All rats were randomly assigned to different groups.

### SNI-induced neuropathic pain model

SNI was performed according to previous methods, with slight modifications; this peripheral nerve injury can simulate mechanical allodynia [32]. Briefly, rats were anaesthetized with 4% sodium pentobarbital. The lateral medial thigh of the left hind limb of the rats was dissected to expose the sciatic nerve and its three branches: the common peroneal, tibial and sural nerves. The tibial and common peroneal nerves were ligated with 4-0 silk and sectioned (removing 2 mm in length), whereas the sural nerve was left intact. The muscle layer and skin layer were sutured separately. The sciatic nerve of the sham-operated group was exposed without ligation or cutting.

### Behavioural testing

The up-down paradigm was performed to measure the 50% PWT in response to mechanical stimulation, as described in previous work [33]. Rats were always acclimated in the measuring case for 15 min before the test. Nine von Frey filaments (0.6, 1, 1.4, 2, 4, 6, 8, 10 and 15 g) were used to measure mechanical allodynia. The 4-g fibre filament was measured first. The filament was applied to the sole with a sufficient force for approximately 8 s. If the rat withdrew its paw or exhibited escape behaviour (noted as X), then the adjacent smaller filaments were selected to continue the test. Conversely, if the rat did not withdraw its paw or exhibit escape behaviour in response to the stimulus (noted as O), the adjacent larger filaments were selected to proceed with the test. The interval between the two measurements was more than 10 s. When the first XO or OX occurred, another four responses were required to complete the test. In total, six strings with an X or O were obtained through testing. The 50% PWT was obtained by inputting the X or O strings into the up-down reader software [34]. If the strings were all X or O, the 50% PWT was assigned 15 or 0.6 g, respectively.

### Intraspinal microinjections

Intraspinal microinjections were slightly modified, according to Kevin et al. [35, 36]. Briefly, rats were anaesthetized and placed in a stereotactic frame, where the spine was fixed. The T13 vertebra was palpated and traced to localize the incision. A 3-cm incision was made in the posterior median line, with T13 as the midpoint, and then the T12–L1 laminae were exposed. The left lamina of T13 was removed to gently expose the spinal cord. A pulled-glass pipette (World Precision Instruments, item no. 1B100F-4) was pulled using a micropipette puller (Sutter Instruments, model P-97) to a tip diameter of 100 μm. The pipette (loaded with a virus or control vector) was attached to an adapter for glass needles (World Precision Instruments, Nanoliter 2010). The injection point was located 0.5 mm to the left of the posterior median sulcus at the level of the L5 spinal cord; the depth of the injection was 0.5 mm from the spinal cord dorsal surface, which reached laminae II to IV. The needle remained in the spinal cord for 10 min after the injection to allow for an equilibration period. Subsequently, the glass pipette was slowly withdrawn. No motor function impairment was observed after the microinjection. SNI surgery was carried out on day 28 after microinjection. Interfering Mlxipl adeno-associated virus (AAV-U6-shMlxipl; titre:3.12^12^ viral genomes (v.g.)/ml) and adeno-associated virus coding Mlxipl (pAAV-CMV-Mlxipl; titre: 3.18^13^ v.g./ml) and control vector were synthesized by the Obio (Shanghai). Microinjection was performed at a rate of 200 nL/min with a pump controller (World Precision Instruments, SYS-MICRO4). One μL of virus or control vectors was deliver to the ipsilateral L4–L6 SDH. The AAV-U6-shMlxipl targeted interference sequence was GCAACTGAGGGA TGAAATA.

### Intrathecal injections

Intrathecal injections were performed according to a method reported by Jasmin and Ohara [37]. Briefly, a 2-cm longitudinal incision was made along the dorsomedial line to expose the L5/L6 spinous process. A PE-10 catheter (Intramedic, USA) was inserted through the incision and advanced 2 cm between the L5 and L6 intervertebral space to reach the lumbosacral enlargement. The correct intrathecal position was confirmed by tail-flick and backflow of cerebrospinal fluid. The catheter was tunneled under the skin, and the end of the catheter was exposed 2 cm outside the skin and fixed to the subcutaneous muscle layer of the neck. On the next day, the rats were injected with 20 μL of 2% lidocaine through the catheter. If lower extremity paralysis occurred within 30 s and was restored within 30 min, the intrathecal catheterization was considered to be successful. Rats with neurologic dysfunction within 3 days post-catheterization were excluded from the study. Small interfering RNA (siRNA), lentivirus coding cJun or their control vectors were intrathecally injected at day 7 after SNI or sham surgery; the injections were carried out once a day for 7 consecutive days. The lentivirus encoding cJun (LvcJun, titre: 3.06^8^ TU/ml) and its control virus (Lv-NC) were synthesized by Saiqing (China). CJun siRNA (sicJun) was purchased from Santa Cruz (cat. no. sc-156028). The volume of intrathecal injection was 20 μL of lentivirus (1 x 10^7^ TU/μL) or sicJun (0.01 nmol/μL).

### Primary microglia culture

Primary microglia were isolated according to the method of Tamashiro et al. [38]. Briefly, postnatal day 2 neonatal rat pups were dissected under aseptic conditions. Both hippocampi were removed and placed in pre-cooled phosphate-buffered saline (PBS). The meninges and blood vessels were carefully removed. After trypsinization, digestion was stopped by adding a complete medium that contained foetal bovine serum. The suspension was evenly triturated, centrifuged and the supernatant was discarded. The cells were gently pipetted into a cell suspension, added to a culture flask at a density of 2 × 10^6^ cells/mL and cultured at 37°C in a 5% CO_2_ incubator. The mixed glial cell culture was incubated for approximately 10 days. To isolated microglia, the flask was shaken at 37°C for 2 h. The cell suspension was then transferred and centrifuged at 1,000 rpm for 5 min. The supernatant was discarded, and the cells were resuspended by adding new medium. Mlxipl or cJun expression in primary microglia was manipulated by transfection of Mlxipl siRNA (Thermo Fisher, AM16708), sicJun (Santa Cruz, USA), lentivirus encoding Mlxipl (Gene Create, China), or LvcJun (Saiqing, China). Each construct was transfected with Lipofectamine 2000 (Invitrogen, USA), according to the manufacturer’s instructions and the experimental design.

### West blot

The rats were anaesthetised, and the L4–L6 SDH (ipsilateral or contralateral) was removed. After adding radioimmunoprecipitation assay (RIPA) lysis buffer with protease inhibitor (Beyotime, China), the SDH was cut, ultrasonically homogenised and lysed on ice for 30 min. The suspension was centrifuged for 15 min at 13,000 rpm, and then the supernatant was removed. The sample concentration was determined with the BCA method. A portion of the protein was diluted in loading buffer and boiled for 10 min in a water bath. The proteins were separated by sodium dodecyl sulphate-polyacrylamide gel electrophoresis (SDS-PAGE). The separated proteins were transferred to a polyvinylidene fluoride (PVDF) membrane (Millipore, USA). After blocking for 1 h at room temperature, the membranes were incubated with primary antibody against Mlxipl (1:800; Abcam, Cat. ab92809, USA), cJun (1:1,000; Cell Signaling Technology, Cat. 9165, USA), p-cJun (1:1,000; Santa Cruz, Cat. sc-822, USA) or GAPDH (1:1,000; Beyotime, Cat. AF1186, China) overnight at 4°C. The membrane was then incubated with the corresponding species of horseradish-peroxidase-conjugated secondary antibody for 1 h at room temperature. The blots were developed with enhanced chemiluminescence (ECL) solution and imaged using an ultra-sensitive chemiluminescence imaging system (Bio-Rad, USA). The intensity of the target protein bands was measured and quantified by ImageJ software (National Institutes of Health, USA) and normalized to the intensity of the GAPDH band.

### Immunofluorescence

The rats were deeply anaesthetized and then intracardially perfused with PBS and 4% cold 4% paraformaldehyde (PFA). After perfusion, the L4–L6 spinal cord was removed, and the surrounding tissues of the spinal cord were dissected away. The spinal cord was post-fixed in 4% PFA for 24 h and then transferred to 30% sucrose until it sank. The spinal cord was embedded in Optimal Cutting Temperature (OCT) compound and axially sectioned with a cryostat at 10 μm. These sections were subsequently processed for immunofluorescence. Rabbit anti-Mlxipl (1:200; Abcam, Cat. ab92809, USA) was incubated with mouse monoclonal anti-cJun (1:300, Cell Signaling Technology, Cat. 9165S, USA), goat polyclonal anti-ionised calcium-binding adapter molecule 1 (Iba1, a microglial marker, 1:800; Abcam, ab5076, USA), mouse monoclonal anti-neuronal-specific nuclear protein (NeuN, a neuronal marker, 1:200; Millipore Bioscience, Cat. MAB377, USA) or mouse monoclonal anti-glial fibrillary acidic protein (GFAP, an astrocyte marker, 1:1,000; Novus Biologicals, Cat. NBP1-05197 USA). After overnight incubation at 4°C, the sections were incubated with secondary antibody conjugated to CY3 (1:400; Jackson ImmunoResearch, USA) or FITC (1:400; Jackson ImmunoResearch) for 2 h at room temperature. Subsequently, the sections were incubated with DAPI for 10 min to stain the nuclei. Finally, an anti-fluorescence quenched sealer was used to cover the sections, and they were examined by confocal microscopy (Nikon Eclipse Ti, Japan). NIS-Elements AR version 4.0 software (Nikon, Japan) was applied for quantitative analysis of immunofluorescence intensity.

### Quantitative real-time polymerase chain reaction (qPCR)

Total messenger RNA (mRNA) was extracted from rat L4–L6 SDH or primary microglia with TRIzol reagent (Takara, Japan). TB Green™ Premix Ex Taq™ II and Prime Script™ RT Master Mix (Takara, Japan) were used for reverse transcription and qPCR. mRNA levels were detected and analyzed with a Bio-Rad thermal cycler. The primers were synthesized by Sangon (China). The specific primers for the target gene are listed in Supplementary Table 1. All results were normalized to GAPDH; the qPCR data were calculated using the 2^-ΔΔCt^ method.

### Enzyme-linked immunosorbent assay (ELISA)

The supernatant of primary microglia treated with lipopolysaccharide (LPS; 1 mg/ul for 24 h) was used to measure the protein levels of inflammatory cytokines, namely tumour necrosis factor alpha (TNFα), interleukin (IL)-6 and IL-1β. Microglia were cultured in 6-well plates (approximately 5 x 10^5^ cells/mL) and incubated with siMlxipl (GENECHEM, Shanghai, China), LvMlxipl (Genecreate, Wuhan, China), sicJun, LvJun or their control vectors for 48 h, followed by incubation with 1 mg/mL LPS for 24 h. After collecting the supernatant, TNF-α, IL-6 and IL-1β levels were determined with ELISA kits (BD Biosciences, USA), following the manufacturer’s instructions. The absorbance of each sample was measured in triplicate at 450 nm.

### Construction of plasmids and dual-luciferase reporters

To generate the pcDNA3.1-cJun vector, we amplified and cloned the cJun coding sequence (CDS) into the pcDNA3.1 vector (Promega, CA, USA) using the *Nhe*I and *Not*I restriction enzymes. The full-length Mlxipl promoter sequence that contained three predicted cJun binding sites—Mlxipl1, Mlxipl2 and Mlxipl3—were amplified and cloned downstream of the firefly luciferase gene into the pGL3 vector with the *Nhe*I and *Xho*1 restriction enzymes. Mutation of pGL3-Mlxipl3 (WT: ATTTACTCT, MUT: CGGGCAGAG) was synthesized by Saiqing (China). For luciferase reporter assays, rat primary microglia were co-transfected with the luciferase constructs described above and the control vector pRL-TK (Promega, CA, USA) with pcDNA3.1-cJun vector or control pcDNA3.1-NC using Lipofectamine 2000 following the manufacturer’s instructions. Subsequently, the luciferase activity was measured using a dual reporter luciferase assay kit (Promega, USA). The relative luciferase activity of the target promoter was represented by firefly luciferase activity, which was normalized by *Renilla* luciferase activity. Three individual transfection experiments were performed. The primer sequences used for PCR amplification of plasmid construction are listed in Supplementary Table 2.

### Chromatin immunoprecipitation (ChIP) assay

ChIP assays were performed by following the Pierce Agarose ChIP Kit protocol (Thermo Scientific, USA). Primary rat primary microglia were fixed with 1% formaldehyde for 10 min. The cells were scraped and then centrifuged to obtain a pellet. The pellet was resuspended with lysis buffer, the chromatin was sonically sheared, and the solution was incubated on ice for 10 min. The lysate was centrifuged at 13,000 rpm for 10 min, and the supernatant was diluted in ChIP dilution buffer. Subsequently, the efficiency of cutting chromatin was detected with a 1% agarose gel. The supernatant was then incubated with anti-cJun antibody overnight at 4°C. Following this incubation, protein A/G was added into the solution, and protein A/G plus agarose was used to collect the immunocomplex. The immunocomplex was washed sequentially with wash buffer. The immunocomplex was eluted with elution buffer and the cross-links were reversed by heating for 4 h at 65°C. DNA was recovered and subjected to PCR amplification using primers specific for the detection of regions that contained the promoter sites. The primers are listed in Supplementary Table 3.

### Statistical analysis

The results are presented as the mean ± standard deviation (SD) for at least three independent experiments. GraphPad Prism Version 7.0 was applied for statistical analyses. Two-way analysis of variance (ANOVA) followed by a post hoc Tukey test was used to compare the differences among groups over time. Student’s *t*-test was used to analyse differences between two groups. A two-tailed *P* < 0.05 was considered statistically significant.

## Supplementary Material

Supplementary Figures

Supplementary Tables
