# Supplementary material for: Upregulation of Mlxipl induced by cJun in the spinal dorsal horn after peripheral nerve injury counteracts mechanical allodynia by inhibiting neuroinflammation
Source: Aging (Albany NY). 2020 Jun 9;12(11):11004–24. doi: 10.18632/aging.103313 (PMC7346034; doi:10.18632/aging.103313)
Supplement: Supplementary Figures [file aging-12-103313-s002..pdf]

SUPPLEMENTARY FIGURES

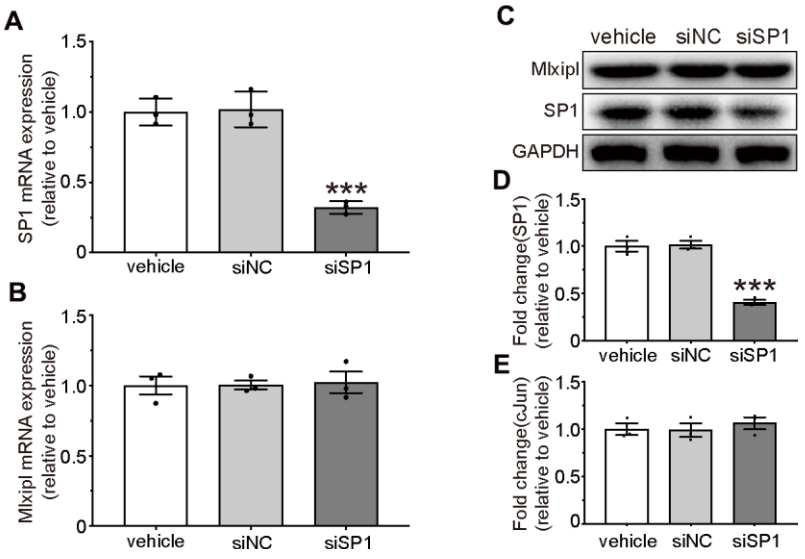

Supplementary Figure 1. Knockdown of SP1 did not significantly inhibit the mRNA and protein expression of Mlxipl. QPCR (**A**–**B**) and western blot (**C**) were performed to detect the expression of SP1 and Mlxipl. Primary microglia were co-transfected with siSP1 or negative control siRNA for 48 hr. Quantification of the western blot (**D**–**E**). Data relative to vehicle. N = 3. ***P < 0.001 vs. vehicle.

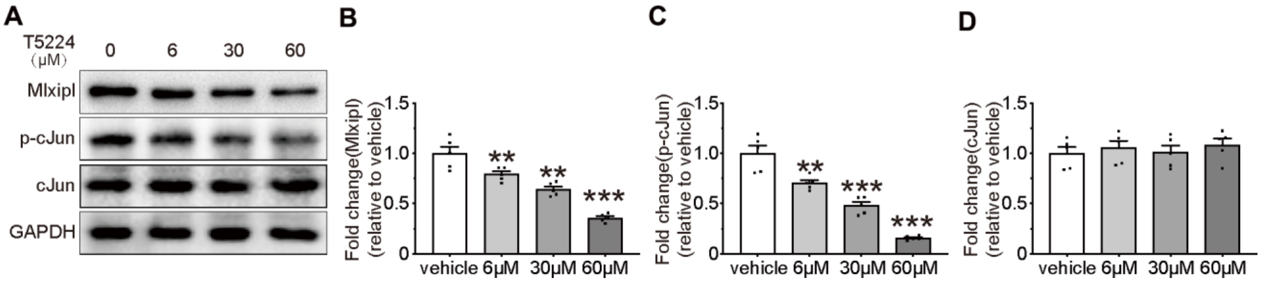

Supplementary Figure 2. T5224 inhibited the expression of p-cJun and Mlxipl. Western blot was performed to detect the expression of target genes. Primary microglia were co-transfected with T5224 for 48 hr. Quantification of the western blot (**D**–**E**). Data relative to vehicle. N = 3. ***P < 0.001, **P < 0.05, *P < 0.01 vs. vehicle.
