# Supplementary material for: Upregulation of Mlxipl induced by cJun in the spinal dorsal horn after peripheral nerve injury counteracts mechanical allodynia by inhibiting neuroinflammation
Source: Aging (Albany NY). 2020 Jun 9;12(11):11004–24. doi: 10.18632/aging.103313 (PMC7346034; doi:10.18632/aging.103313)
Supplement: Supplementary Tables [file aging-12-103313-s001..pdf]

SUPPLEMENTARY TABLES

Supplementary Table 1. Primer set list for qPCR.

Target gene	Direction	Primer sequence
Mlxipl	Forward	GGGTGCCCATCACACATCAG
	Reverse	TGCGGTAGACACCATCCCAT
cJun	Forward	GGCACATCACCACTACACCG
	Reverse	GTGACACTGGGCAGCGTATT
TNF-α	Forward	GCATGATCCGAGATGTGGAACTGG
	Reverse	CGCCACGAGCAGGAATGAGAAG
IL-1β	Forward	ATCTCACAGCAGCATCTCGACAAG
	Reverse	CACACTAGCAGGTCGTCATCATCC
IL-6	Forward	AGGAGTGGCTAAGGACCAAGACC
	Reverse	TGCCGAGTAGACCTCATAGTGACC
GAPDH	Forward	ACAGCAACAGGGTGGTGGAC
	Reverse	TTTGAGGGTGCAGCGAACTT

Supplementary Table 2. Primer for construction of plasmids and dual-luciferase reporters.

Target gene	Direction	Primer sequence (restriction enzymes in red)
Mlxipl-promoter	NheI-F	AAAAGCTAGCACAAGAATGACAATAGACAGCCAAGG
	Xho1-R	AAAACTCGAGGGATAGCAGTTTGAGAGCCAATC
cJun CDS	Nhe1-F	AAAAGCTAGCATGACTGCAAAGATGGAAACGA
	NotI -R	AAAAGCGGCCGCTCAAAACGTTTGCAACTGCTG

Supplementary Table 3. Primers for the target promoter fragments for ChIP-qPCR.

Target gene	Direction	Primer sequence (restriction enzymes in red)
Mlxipl1-promoter	Forward	ACTTCATCACATCACGCCTAACC
	Reverse	GAAGTTTTATGGTCACGGCTTCA
Mlxipl2-promoter	Forward	ATAAGTGAGACCCCCCCCAT
	Reverse	TCTTTGTTTAGTTTCCCTCCATGTC
Mlxipl3-promoter	Forward	CTCGTGTTTATGTGTCTAGGAAACTTATG
	Reverse	GCCGTCAAGTAGATGGCTAGCT
GAPDH	Forward	CATGGGTGTGAACCATGAGA
	Reverse	GTCTTCTGGGTGGCAGTGAT

Supplementary Table 4. List of cJun prediction transcription factor.

Bioinformatics sites	prediction transcription factor	Bioinformatics sites	prediction transcription factor	Bioinformatics sites	prediction transcription factor
JAPSAR	NFYA	Promo	GR	GTRD	Mbd3
	Foxq1		C/EBPbeta		Tcf7l2
	Foxd3		C/EBPalpha		Sox10
	Cebpa		USF2		Pou1f1
	FOS::Jun		USF-1		Jun
	Ddit3::Cebpa		AP-2		Hnf4a
	SP1		c-Fos		Egr2
	FEV		C/EBPdelta		Sp1
	NFATC2		NF-1		Nr1h4
	NR4A2		Nkx2-1		Mlxipl
	Foxa2		SRF		Chd8
	Znf423		HNF-3beta		
	SOX10		FOS::Jun		
	Gfi1		DBP		
	CREB1		HNF-3alpha		
	Mafb		CREB		
			CBF(2)		
			Crx		
			HNF-1		
			Sp1		
